# Numerical Investigation of the Formation of a Failure Cone during the Pullout of an Undercutting Anchor

**DOI:** 10.3390/ma16052010

**Published:** 2023-02-28

**Authors:** Józef Jonak, Robert Karpiński, Andrzej Wójcik, Michał Siegmund

**Affiliations:** 1 Department of Machine Design and Mechatronics, Faculty of Mechanical Engineering, Lublin University of Technology, Nadbystrzycka 36, 20-618 Lublin, Poland; 2 KOMAG Institute of Mining Technology, Pszczyńska 37, 44-100 Gliwice, Poland

**Keywords:** undercut anchor, failure cone, FEM simulation, radial cracks

## Abstract

Previously published articles on anchors have mainly focused on determining the pullout force of the anchor (depending on the strength parameters of the concrete), the geometric parameters of the anchor head, and the effective anchor depth. The extent (volume) of the so-called failure cone has often addressed as a secondary matter, serving only to approximate the size of the zone of potential failure of the medium in which the anchor is installed. For the authors of these presented research results, from the perspective of evaluating the proposed stripping technology, an important aspect was the determination of the extent and volume of the stripping, as well as the determination of why the defragmentation of the cone of failure favors the removal of the stripping products. Therefore, it is reasonable to conduct research on the proposed topic. Thus far, the authors have shown that the ratio of the radius of the base of the destruction cone to the anchorage depth is significantly larger than in concrete (~1.5) and ranges from 3.9–4.2. The purpose of the presented research was to determine the influence of rock strength parameters on the mechanism of failure cone formation, including, in particular, the potential for defragmentation. The analysis was conducted with the finite element method (FEM) using the ABAQUS program. The scope of the analysis included two categories of rocks, i.e., those with low compressive strength (<100 MPa) and strong rocks (>100 MPa). Due to the limitations of the proposed stripping method, the analysis was conducted for an effective anchoring depth limited to 100 mm. It was shown that for anchorage depths <100 mm, for rocks with high compressive strength (above 100 MPa), there is a tendency to spontaneously generate radial cracks, leading to the fragmentation of the failure zone. The results of the numerical analysis were verified by field tests, yielding convergent results regarding the course of the de-fragmentation mechanism. In conclusion, it was found that in the case of gray sandstones, with strengths of 50–100 MPa, the uniform type of detachment (compact cone of detachment) dominates, but with a much larger radius of the base (a greater extent of detachment on the free surface).

## 1. Introduction

**Figure 1:**
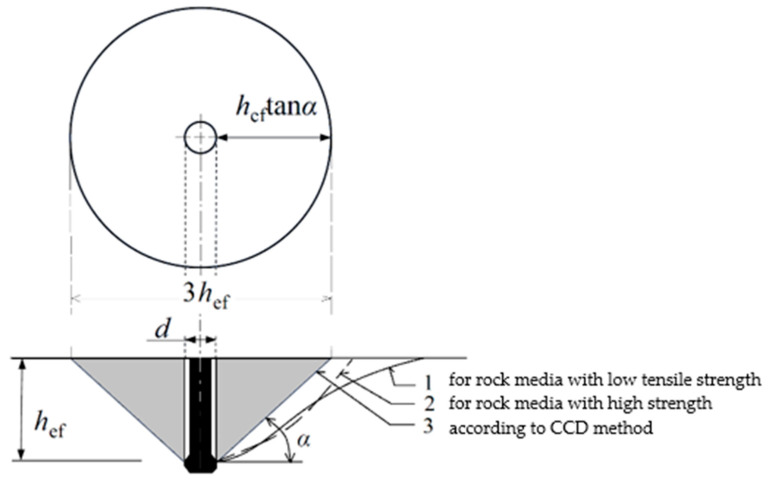
Formation of the failure zone of the medium under the action of the undercutting anchor, where: d—diameter of anchor hole, *h*_ef_ —effective anchor depth, and α—angle of failure cone according to CCD method (α = ~35°).

Undercutting anchors are mainly used in equipment used for embedding steel structures in engineered concrete constructions [1,2,3,4]. The authors of this study, on the other hand, have used undercutting anchors for rock mass detachment in their previous studies [5,6]. This is an unconventional method of detachment, which may have applications in special situations occurring, for example, in mining operations (excavation of rescue pits, etc.) [7,8]. Previous studies on the subject have focused mainly on the development of theoretical models of the impact of anchors on the concrete medium, as well as the practice of the installation of the anchors under consideration [9,10]. The two most notable models are the traditional 45° cone approach (ACI Method 349-85) and the concrete capacity design (CCD) method [11,12], where, in the latter, the angle of the failure surface (cone or pyramid) is 35° [13]. The potential failure of the medium in which the anchor is fixed (which occurs when the load capacity of the attachment is exceeded) is approximated by the so-called cone of failure [14,15] (with a given angle of formation, as in Figure 1); alternatively, as in more recent recommendations, this form of failure is approximated by a pyramid. In fact, the shape of the potential failure zone resulting from a single anchor pull-out is simplified to a four-sided pyramid failure surface [16,17].

In existing design guidelines, the failure zone of the medium is approximated by a cone with a cone angle a equal to *α* = 35° [14]. This is a convenient approximation when one is mainly interested in the load capacity of the anchor (the force that causes it to pull out). The maximum value of the anchor pullout force occurs for a length of the gap opening (measured along the cone’s formation) equal to about 0.45 of the destruction cone’s formation length. The further development of the fracture takes place alongside a decrease in the pulling force and is not usually subjected to closer scrutiny. The destruction model adopted in this way significantly limits the ability to estimate the potential volume of the stripped blocks, which is of interest to the authors because of the performance estimates of the proposed detachment technology. Detailed studies on the formation of the failure zone during anchor pullout carried out by, for example, [18,19], as well as the authors of [20,21,22,23], show that the failure zone significantly differs in its shape from the failure cone adopted in standard studies. Depending on the strength of the medium, the trajectories of the fractures leading to the detachment of the solid (cone-like) depend significantly on the solid’s tensile strength and the proportion of fracture energy in so-called Mode I and Mode II. To simplify the issue, for high-strength materials, the detachment surface assumes a shape similar to a paraboloid (curve 2, Figure 1), while for low-strength media, the course is more complex [5]. In the latter case, in the first phase of progression, the fracture penetrates at a small angle, along a parabola, and, at some point, transitions into a trajectory along a hyperbola. This results in a significant increase in the extent of detachment measured from the anchor axis along the free surface of the medium [21]. In both cases (curve 1,2—Figure 1), the extent of detachment (failure zone) is significantly greater (two to three times, e.g., [20]) than what is implied by the failure zone models recommended by the standard [14]. In contrast, [24] analyzed the effect of the diameter of the undercutting anchor head on the potential extent of the failure zone of the rock medium. For a fixed effective anchor depth and an equal angle of the conical undercutting anchor head, no significant effect of this parameter on the formation of the extent of the failure zone of the rock medium was found.

Another aspect that has been reported in previous studies [25,26] is the possibility of so-called radial gaps, which promote the separation of the destruction zone (destruction cone) into smaller fractions. However, the mechanism of their formation is not clear, and considering the research conducted so far, it is assumed that the microcracks existing in a given rock medium or potential layering or faulting are mainly responsible for their formation.

From the perspective of researching the proposed detachment technology, the potential presence of radial fractures is an important element, as it can facilitate the removal of detached blocks from the work zone of rescue crews, without the need for additional grinding.

On the other hand, in the numerical analyses of the issue conducted so far, which have been carried out using the FEM program ABAQUS as well as plane and, especially, axisymmetric models, it was not possible to clarify this aspect further, so an attempt was made to explore the issue of the potential generation of radial fractures (based on 3D FEM models) cause by the undercutting anchor’s interaction with the rock medium.

The previously published articles on the subject were mainly limited to determining the maximum force that can be applied to an anchor so that it does not pull out, and determining the point at which such force occurs. The extent of the break-out (failure cone) was treated as a secondary matter, serving only to approximate the size of the zone of potential material failure. For the authors of the presented research results, this is a very important aspect from the perspective of predicting the volume of potentially detached rock and the actual form of detachment. This is due to the limitations of the space available for the removal of breakout products, as well as the load capacity of the available transport equipment, in the specific conditions of the application of the proposed breakout technology (such as mining or construction disasters and rescue operations, e.g., collapsed buildings after an earthquake) [27,28]. This justifies the undertaking of research on the reported subject.

In conclusion, it should be said that, given the current state of knowledge, the problem of the formation and development of radial fractures in rocks in the zone of the cone of destruction is an issue that has not yet been adequately clarified. In view of the importance of this aspect to the proposed detachment technology as well as the use of undercutting anchors in fastening technology, broader analysis is required to justify the addressal of such a topic.

The purpose of the proposed study was to determine the mechanism of the propagation of the failure zone under the action of the undercutting anchor. The study was carried out in the context of the potential formation and development of radial fractures leading to the separation of the failure cone into smaller fractions. Analyses were performed in continuous media, undisturbed by cracks or faulting, with different combinations of mechanical parameters of the medium (tensile strength, fracture energy, and the Coulomb friction coefficient between the surface of the anchor head and rock).

The results of these analyses are presented in the following section. 

## 2. Materials and Methods

### 2.1. Assumptions for Simulation

The strength parameters of the rock medium used in the model are adequately similar to those determined for rocks subjected to anchor pull-out tests under field conditions, which was the subject of discussion in the authors’ previous studies [29,30].

The mechanical parameters of the materials used in the simulation of the rock model and the anchor are shown in Table 1 and Table 2. In the presented experiment, the modeled base material was sandstone.

The criteria specified in the FEM models were damage criterion—“max. principal stress”—and damage evolution—“softening linear”; the medium was continuous and homogeneous.

### 2.2. Geometry of the Model 

**Figure 2:**
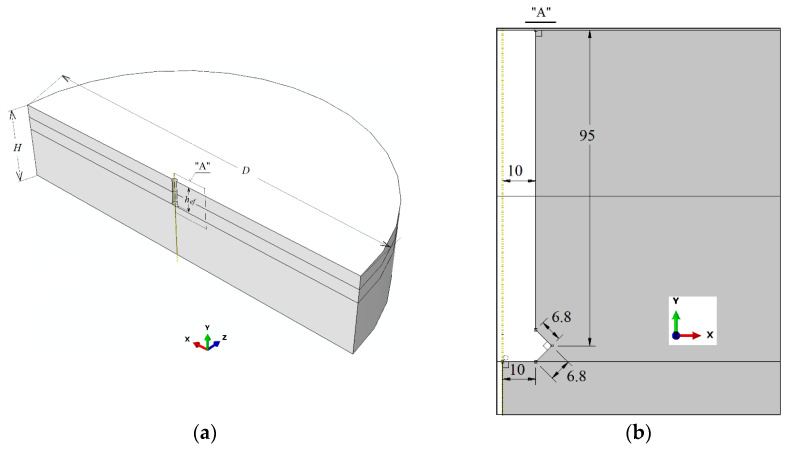
Geometry of the rock medium model, including the undercut for the anchor: *D*—diameter, *H*—height of the rock model, and *h*_ef_—effective anchor depth; (**a**) half model; (**b**) the dimensions of the anchor undercut.

The 3D geometric model of the rock medium was designed in the shape of a cylinder. Due to the symmetry of the model and the way in which cross-sections are represented in ABAQUS, half of a cylinder (the plane of symmetry includes the axis of the cylinder) with dimensions corresponding to cylinder diameter *D* = 1400 mm and height *H* = 300 mm was used for calculations. Thus, a 3D geometric model was used in the analysis, as in Figure 2a. Meanwhile, the dimensions of the hole and the undercut in the rock for the anchor are shown in Figure 2b (with detail “A” of Figure 2a enlarged). The effective anchor depth was assumed to be equal to *h*_ef_ = 95 mm. The large *D*/*h*_ef_ ratio avoids the influence of supports, which is characteristic of the pull-out method.

### 2.3. Boundary Conditions

**Figure 3:**
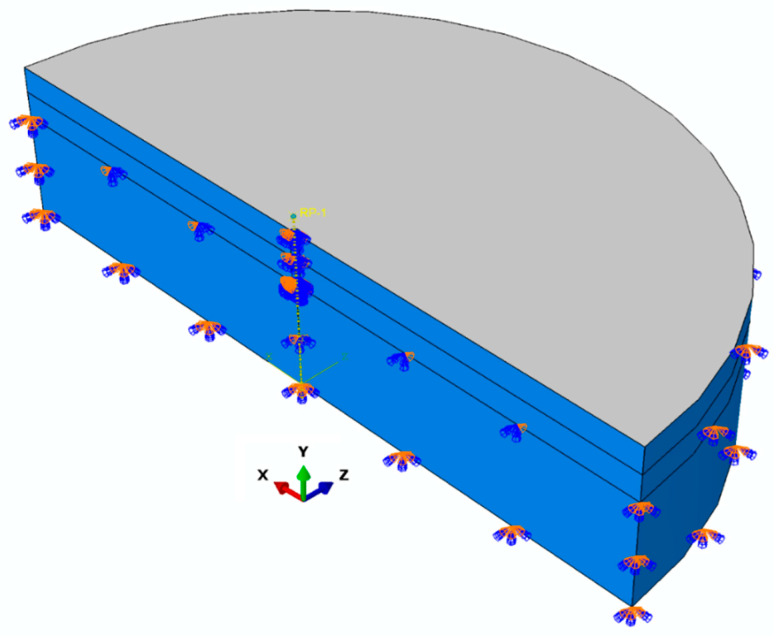
Method of restraining the considered model of the rock medium.

Restraint was used: in the base of the model (cylinder) and the side walls up to the height of the hole, the nodes of the model were deprived of three translational degrees of freedom, that is, U1 = U2 = U3 = 0 (as in Figure 3). The proposed type of restraint results from the fact that the adopted model of the medium is a section of the half-space of the rock medium. The dimensions of this model were assumed to be so large that the restraints of the nodes of the model lack the influence of the stress field generated by the anchor and the support of the anchor puller. U1, U2, and U3 correspond to translations along the axis of the adopted coordinate system.

According to the CCD procedure, for *h*_ef_ = 95 mm, the potential extent of detachment measured on the free rock surface was ~1.5*h*_ef_ = 1.5 × 95 mm = 142.5 mm. Therefore, it was much smaller than the assumed radius of the model, which is equal to 700 mm (potential lack of influence of restraints on the propagation of the destruction zone). 

**Figure 4:**
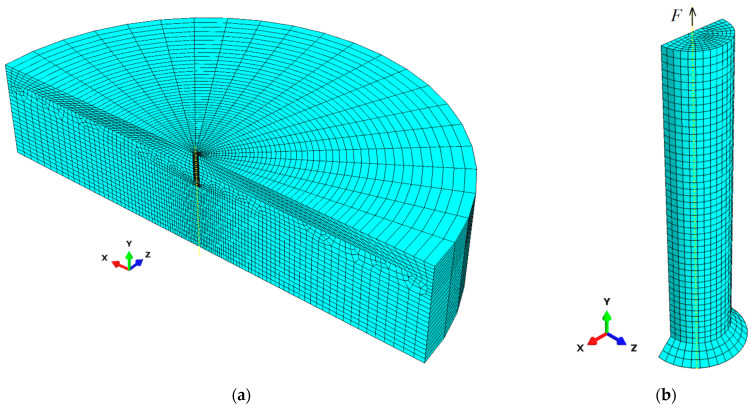
Finite element mesh for models of: (**a**) rock medium, (**b**) anchor.

The analysis was conducted for the assumed forcing of the anchor movement along the Y axis of the adopted coordinate system (with the load applied to the upper surface of the anchor, along the Y axis, as in Figure 4b). Using ABAQUS routines, a mesh of hexagonal elements with a “sweep” arrangement was used in the model. C3D8R elements—eight-node linear elements with reduced integration—were used to build the mesh. As a basic example, elements with global linear dimensions of 14 mm were used. To reduce the complexity of the computational tasks, radially, the size of the elements increases outward from 5 mm to 30 mm. The model of the rock medium and anchor, discretized with finite elements, is illustrated in Figure 4.

The problem was implemented as a contact one; a surface-to-surface contact was assumed with properties in the normal direction of the hard contact type and in the tangential direction of the penalty contact type, with a friction coefficient of Coulomb *µ* = 0.6. 

## 3. Results

### 3.1. Results of Sensitivity Analysis of the Model to the Size of the Finite Elements

The fracture analysis was carried out using the XFEM algorithm, which, according to many sources (e.g., [31]), is not very sensitive to the size of the finite element mesh elements of the model under consideration; however, in some fracture mechanics problems, it is worth carrying out a so-called sensitivity analysis of the FEM model to determine its sensitivity to the size of the finite element mesh in order to select its optimal structure.

In order to determine the sensitivity of the model to the size of the finite elements of the FEM mesh, a broader analysis of this aspect was carried out, using relevant models.

#### 3.1.1. Model “A”

**Figure 5:**
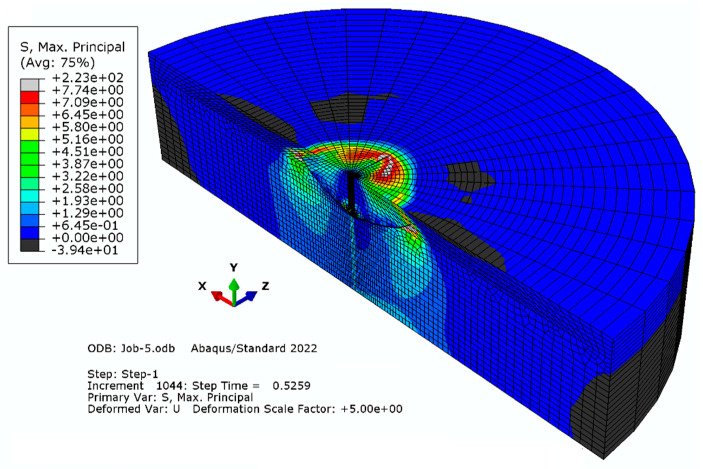
Simulation result for mesh model “A”.

Elements with a global linear dimension of 14 mm were used; radially, the size of the elements increases outward starting from 5 mm to 30 mm. The number of elements on the half-diameter of the model was 22. The characteristics of the finite element mesh of the model were as follows: the number of nodes—28877 and the number of elements—26086. The result of the simulation (the distribution of maximum stress (max) and the trajectory of the crack) is illustrated in Figure 5.

#### 3.1.2. Model “B”

**Figure 6:**
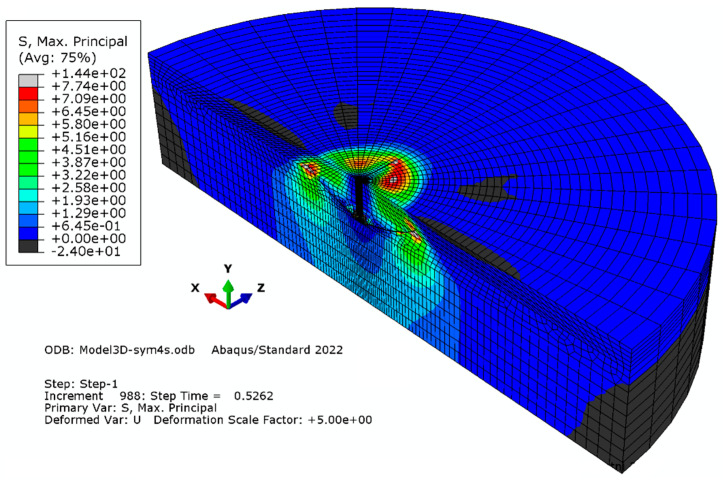
Simulation result for mesh model “B”.

Elements with a global linear dimension equal to 28 mm were used; radially, the size of the elements increases outward from 5.6 mm to 28.2 mm. The number of elements on the half-diameter of the model totaled 22. The characteristics of the finite element mesh of the model are as follows: the number of nodes—23856; the number of elements—21280 (as illustrated in Figure 6).

#### 3.1.3. Model “C”

**Figure 7:**
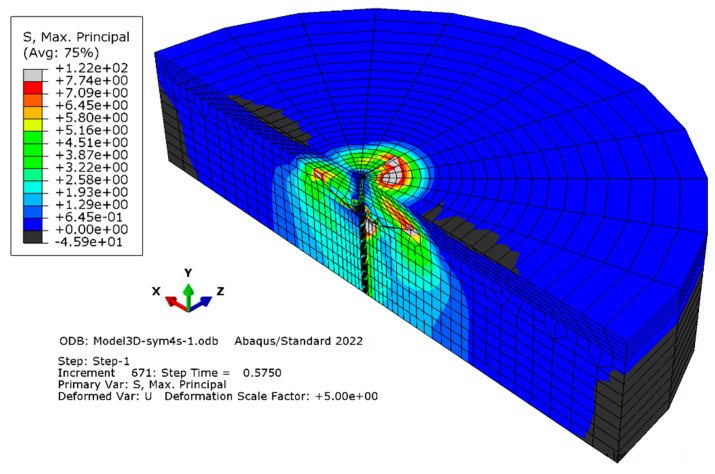
Simulation result for mesh model “C”.

Elements with a global linear dimension of 28 mm were used; radially, the size of the elements increases outward from 11.5 mm to 57 mm. The number of elements on half the diameter of the model corresponded to 12. The characteristics of the finite element mesh of the model are as follows: the number of nodes—5450; the number of elements—4530 (Figure 7).

#### 3.1.4. Model “D”

**Figure 8:**
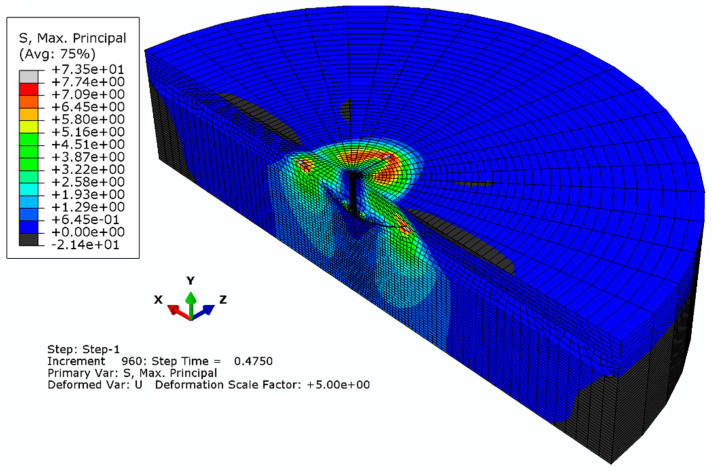
Simulation result for mesh model “D”.

Elements with a global linear dimension of 7 mm were used; radially, the size of the elements increases outward from 5.6 mm to 28.2 mm. The number of elements on the half-diameter of the model corresponded to 22. The characteristics of the finite element mesh of the model are as follows: the number of nodes—46645; the number of elements—42886 (Figure 8).

#### 3.1.5. Model “E”

**Figure 9:**
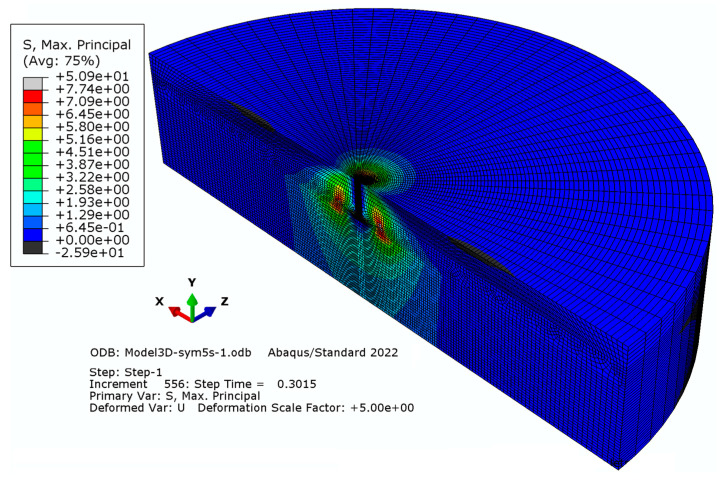
Simulation result for mesh model “E”.

Elements with a global linear dimension of 7 mm were used; radially, the size of the elements increases outward from 5.5 mm to 14.8 mm. The number of elements on half the diameter of the model corresponded to 44. The characteristics of the finite element mesh of the model are as follows: the number of nodes—190260; the number of elements—180474 (Figure 9).

#### 3.1.6. Model “F”

**Figure 10:**
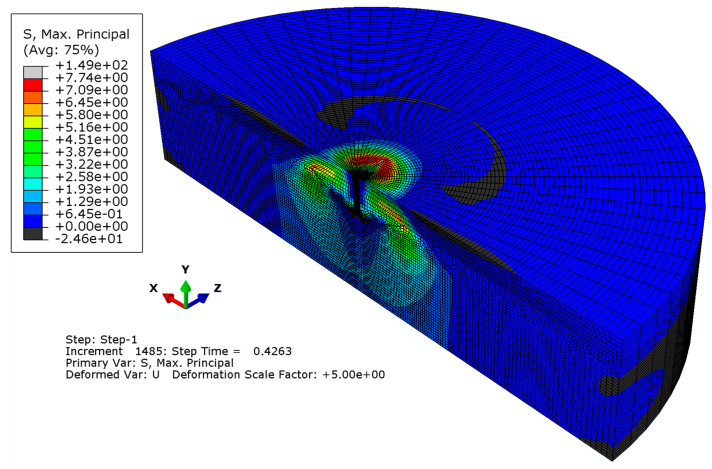
Simulation result for mesh model “F”.

Elements with a global linear dimension of 7 mm were used; radially, the size of the elements increases outward from 4.6 mm to 18 mm. The number of elements on half the diameter of the model corresponded to 33. The characteristics of the finite element mesh of the model are as follows: the number of nodes—164242; the number of elements—155502 (Figure 10).

**Figure 11:**
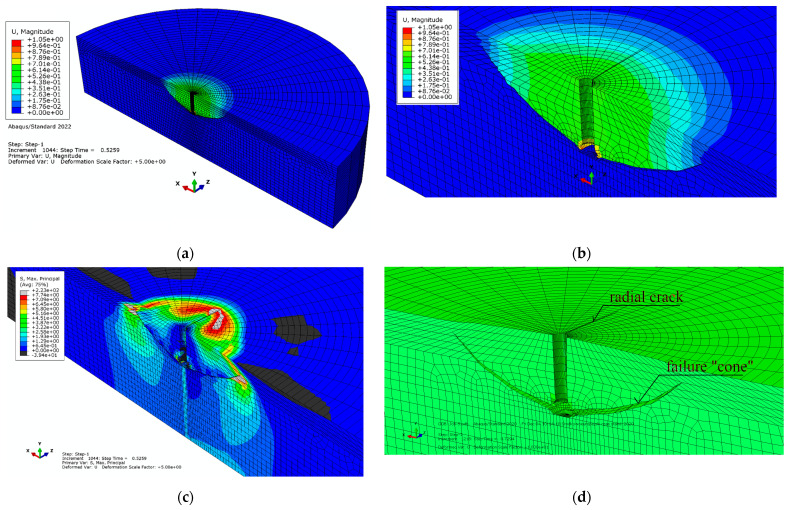
(**a**,**b**) Displacement distribution in the failure zone (in the Y-axis direction); (**c**) max normal stress distribution *σ*_max_ in the failure “cone”; and (**d**) development of the failure “cone” surface and radial gap.

**Figure 12:**
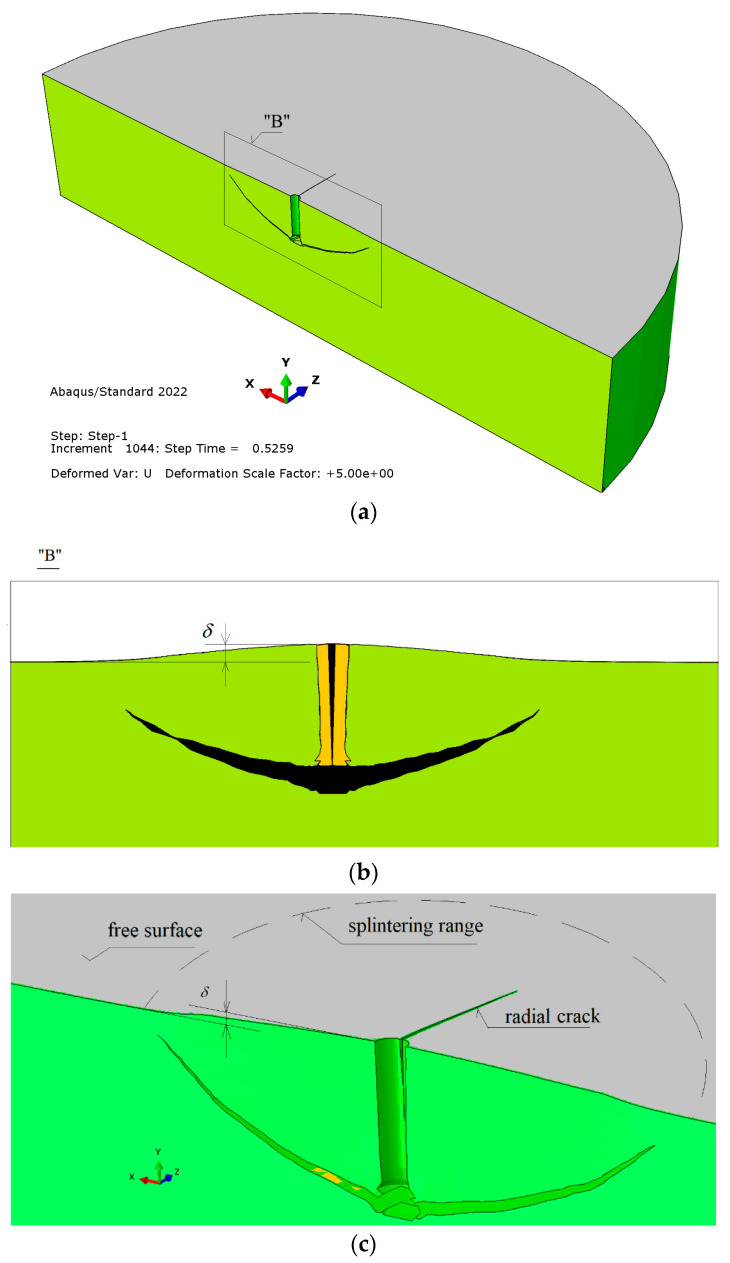
Propagation of the failure zone, radial fracture (**a**,**c**), and deformation of the rock medium -*δ*, each measured relative to the free surface of the model (**b**,**c**).

As a result of the analysis, it was found that the density of the finite element mesh has only a negligible effect on the formation of the trajectory of the resulting crack. However, it significantly affects the calculation time and the smoothness of the surface of the failure cone. As a consequence of the analysis, a research finite element mesh model of type “A” was selected for further study, where elements with a global linear dimension of 14 mm were used to build the model, and to reduce the computational task, radially, the size of the elements increases outwardly along the radius, starting from 5 mm to 30 mm. The results obtained from the detailed analysis of the issue are illustrated in Figure 11 and Figure 12.

Figure 11a,b show the distribution of resultant displacements in the failure zone and the resulting outline of the failure zone (the so-called failure cone). It can be seen from these figures that the largest displacements of the rock medium occur at the periphery of the anchor hole and decrease along the radius of the model. In addition, the appearance of a radial crack was noted in a plane almost perpendicular to the plane of symmetry of the model.

Figure 11c, in turn, shows the distribution of max normal stresses *σ*_max_. A particular concentration of stresses can be seen near the top of the cracks; the radial one is particularly notable. Figure 11d illustrates the trajectories of the resulting cracks against the background of the model mesh (in an enlarged deformation scale).

To better illustrate the obtained relationships, Figure 12 shows (without FEM mesh) the obtained outline of the failure surface (Figure 12a,b), the propagation of the radial crack (Figure 12a,c), and the image of the deformation of the rock medium under the action of the anchor, which is observed in a cross-section through the axis of the anchor hole (Figure 12b).

### 3.2. Effect of Changing the Mechanical Parameters of the Rock Medium

**Figure 13:**
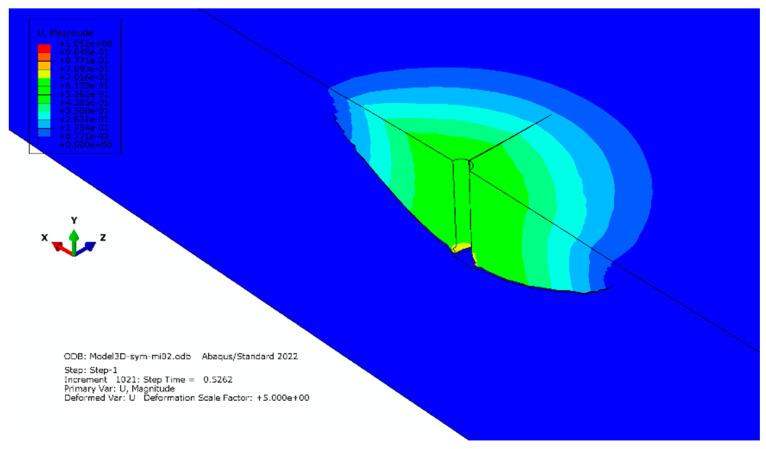
The course of deformation of the rock material under the action of the undercutting anchor, the outline of the zone (cone) of destruction, and the development of the radial crack for the coefficient of friction of the anchor with the rock (*µ* = 0.2).

**Figure 14:**
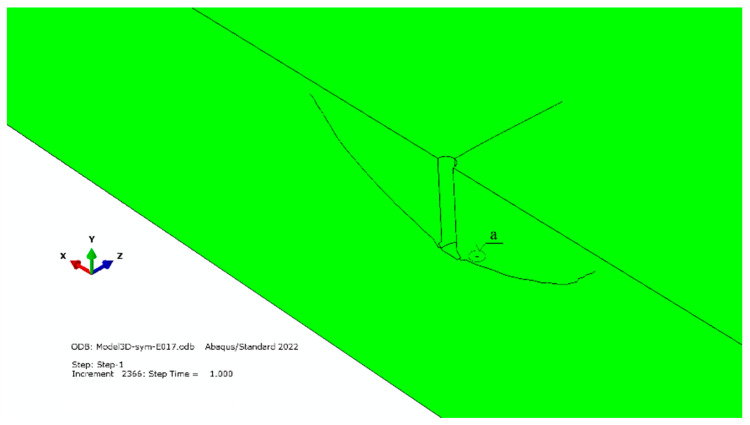
Appearance of an additional gap in the anchor head area, for *G*_fc_ = 0.355 N/mm.

**Figure 15:**
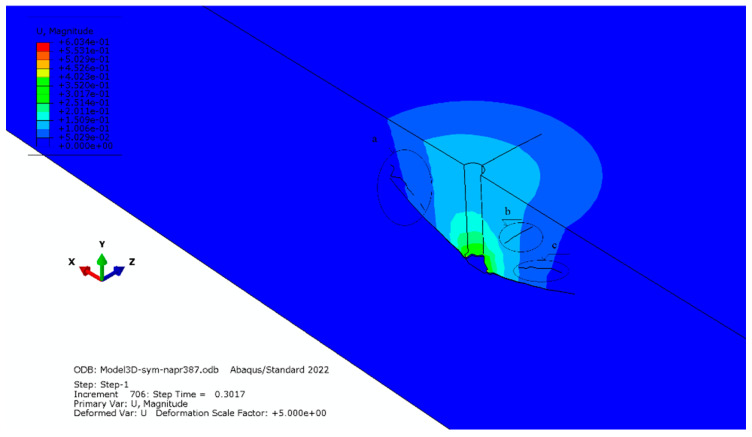
Concentration of micro cracks developing in the anchor head impact zone.

**Figure 16:**
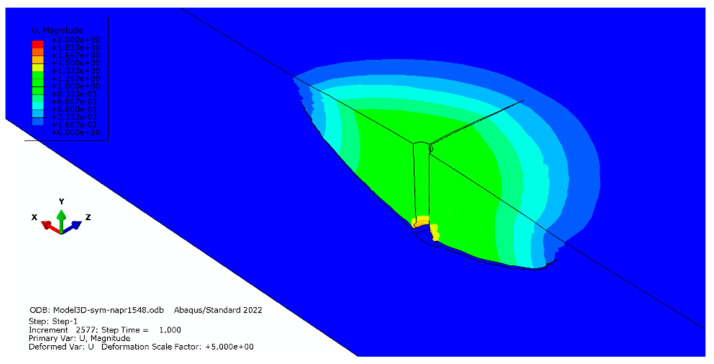
The course of deformation of the medium and propagation of the destruction zone and radial gap for (*f_t_* = 15.48 MPa).

In order to extend the scope of the conducted analysis, the influence of selected mechanical parameters of the rock medium on the potential occurrence of radial fractures (cracks) in the zone of formation of the so-called failure cone was analyzed. The influences of the Coulomb friction coefficient (*µ*, in the contact zone of the anchor surface with the rock), tensile strength (*f*_t_), and critical fracture energy rate (*G*_fc_) were taken into account. The results obtained are illustrated in Figure 13, Figure 14, Figure 15 and Figure 16.

Figure 13 shows the results of a simulation carried out with a significantly reduced value of the Coulomb friction coefficient (*µ* = 0.2). The smaller value of this coefficient than in the simulations shown in Figure 11 and Figure 12 (*µ* = 0.6) did not yield a significant change in the behavior of the failure zone.

Figure 13 (as well as Figure 15 and Figure 16) illustrates the deformation of the model obtained in ABAQUS program based on normalized measures of the eigenvectors of the model nodes. For the standard deformation scale of the model used (zoom × 1), the model shows trends in the deformation of the model areas and maps the cracking trajectory of the medium. Contrary to the other images (where zoom >> 1), however, the model does not allow for the depiction of the opening of the gap. 

In Figure 14, contrary to the other images, the scale of displacement is 1:1 (zoom = 1, which is the standard in the program). As a result, only the trajectory of the penetrating crack is visible, and the opening of the crack is not visible. To assure identical simulation conditions, the use of a reduced value of critical fracture energy rate *G*_fc_ (*G*_fc_ = 0.17 N/mm) in place of the one previously used in the simulation (*G*_fc_ = 0.355 N/mm) resulted in conditions that were conducive to the formation of an additional fracture in the anchor head area (Figure 14, detail “a”).

The most remarkable changes in the fracture pattern of the rock medium were found in the case of its significantly reduced tensile strength, as shown in Figure 15. *f_t_* = 3.87 MPa was used in place of the previously used tensile strength of the rock (*f_t_* = 7.74 MPa). As a result, areas appeared (detail “a”, “b” and “c” in Figure 15) with a concentration of micro cracks developing in the anchor head impact zone. 

In contrast, despite the significant increase in the simulation time, the tensile strength of the rock (*f_t_* = 15.48 MPa) in place of that used previously (*f_t_* = 7.74 MPa, Figure 11) did not result in a significant change in the development of the failure zone (Figure 16).

The analyses carried out by both the authors of the study and other researchers showed that for flat and axially symmetrical models, it is not possible to rationally explain the mechanism of the formation and propagation of radial cracks, which are of interest to the authors of the study. There were indications as to their potential existence, but the mechanism of propagation was not thoroughly understood. Figure 15 confirms these suggestions and illustrates crack nucleation and crack development, including radial cracks, showing the mechanism of destruction of the medium’s structure. So far, this process has not been demonstrated in such a way.

## 4. Experimental Verification 

**Figure 17:**
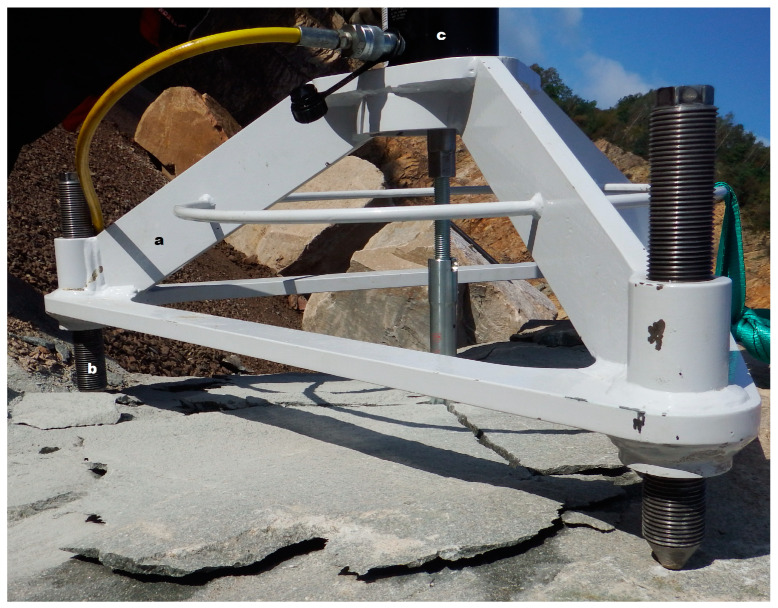
The main components of the mobile anchor-pulling station: (**a**) frame, (**b**) adjustable support, and (**c**) hydraulic cylinder.

Field tests were carried out at the “Braciszów” and “Zalas” mines [21], among others, using a dedicated mobile measuring station to pull out the anchors. The stand consisted of a frame with a socket for attaching a hydraulic cylinder (Figure 17), which was supported at three points; a hydraulic cylinder; a hydraulic pump with a pressure sensor; and a measuring computer.

The stand allows for the measurement of the pull-out force of the anchor. Unlike the instruments dedicated to the implementation of the “Pull-out” test, the radius of the distribution of supports was selected to eliminate the influence of the supports on the distribution of stresses in the anchor’s zone of influence, and to allow for the undisturbed development of the failure zone, with the full detachment of the so-called failure cone, on the free surface of the rock.

**Figure 18:**
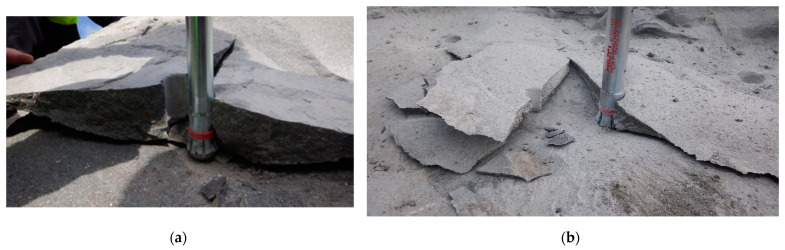
Occurrence of radial fractures during rock block detachment, anchor M20 × 250/100: (**a**) “Braciszów” mine—sandstone; *h*_ef_ = 75 mm. (**b**) “Zalas” mine—porphyry; *h*_ef_ = 55 mm.

In the “Braciszów” mine, the anchor was embedded (and pulled out) in sandstone with a uniaxial compression strength of *f_c_* = 97.4 MPa and a tensile strength of *f_t_* = 6.2 MPa. At the “Zalas” mine, the anchor was embedded (and pulled out) in porphyry with a uniaxial compressive strength of *f_c_* = 106.5 MPa and a tensile strength of *f_t_* = 5.9 MPa. Examples of the results of the stripping of the rock solids are illustrated in Figure 18.

Closer analysis of the field results showed that in addition to the predominant detachment forms of the rock masses in a uniform cone-like form [5,6,7,8,29], there are also disjointed forms, as in Figure 18.

The above forms of failure zone reflect very well the failure mechanism observed in the numerical analysis (e.g., Figure 12). Figure 18a clearly illustrates the existence of radial cracks with a course similar to that observed in the numerical analysis, positively verifying the results of this analysis.

Field tests (Figure 18) confirmed that the loosening of hard rocks with high tearing strength and high *G_fc_* fracture energy with the undercut anchor proceeds rapidly, with radial fractures appearing each time. Ultimately, this leads to the obligatory fragmentation of the “cone of destruction”. This is a clear confirmation of the results of the FEM numerical analysis (Figure 14), where a similar failure mechanism was found. In such a case, the impact of the anchor causes a slight growth of micro-cracks within its head and a very rapid development of the surface of the “cone” of damage, as well as a rapid expansion of the radial crack, leading to detachment according to curve “1” (Figure 1) alongside the rapid (almost explosive) obliteration of the cone of damage into smaller fractions. In the case of rocks of low and medium strength and relatively low fracture energy (e.g., “Brenna” mining), field research [21] showed that the process of generating the failure cone is passive, with the intensive development of micro-cracks in the area of action of the anchor head and the slow development of the surface of the “cone” of destruction (the course according to curve “2” (Figure 1). The mechanism of weak rock destruction observed in field conditions positively verifies the results of the numerical analysis presented in Figure 15, wherein the rapid generation of micro-cracks in the area of the action of the anchor head in the initial stage of the head load was clearly observed. Unlike in hard rocks, the process of the formation of the “cone” of failure is slow, leading to a large degree of loosening on the free surface of the rock medium. At the same time, the deformation of the rock (*δ*, Figure 12) in the case of compacts, which was observed in field tests, was significantly smaller than in rocks of low strength (of the gray sandstone type, as in “Brenna” mining). 

## 5. Discussion

Numerical modeling using methods such as the finite element method (FEM) [32,33,34,35,36], boundary element method (BEM) [37,38], and the application of artificial intelligence (AI) methods [39,40,41,42,43,44] is very widely used in engineering sciences, especially for analyzing the behavior of materials and structures [45,46,47,48]. These methods, combined with experimental studies, enable research into understanding the actual behavior of engineering structures in an effort to achieve their further optimization [49,50,51]. The research conducted to date has mainly focused on analyses leading to an understanding of concrete failure mechanisms [52,53,54,55], the development of methods for estimating anchor load capacity [56,57,58], the effect of anchor design on the pullout force [18,59], and the influence of the technological parameters of anchorage systems [60,61] on the ability of anchors to carry specific loads, including the extent of the breakout area on the free surface of concrete. The results of the analysis showed that in homogeneous materials, it is possible to generate a radial fissure that divides the zone of destruction (cone of detachment) into smaller fractions. The analysis shows that such a crack is oriented perpendicularly to the axis of the considered rock model. Compared to previous results obtained based on partial FEM models [14,18,19], the currently presented results provide new information as to the potential development of the failure zone under the action of the undercutting anchor. So far, it has been suggested that the detachments have a predominantly mono-particle form similar to the cone of destruction [5,8,62], as illustrated in Figure 1, and that the possible separation of the zone of destruction into finer fractions (observed in field studies [21]) is the result of potential disturbances in the homogeneity or continuity of the structure, which are naturally occurring in rock media. The results of the analysis presented herein are consistent with those presented in the literature. According to a number of sources (e.g., [19,63]), there is a potential or the occurrence of radial gaps due to stress distribution (tension) in the upper part of the model in the area of the anchor hole (in the free surface zone), but this aspect was not analyzed further. The present analysis further showed that the lower tensile strength of the rock promotes the generation of a number of micro cracks in the area of the undercutting head. The results of the analysis in this area are consistent with a number of studies [64,65,66,67,68], which, for example, noted that pulling out anchors with small head diameters leads first to the local crushing of the concrete (micro-cracking) under the head and, only at a later stage, to the detachment of a larger element (the so-called cone of failure). It was also found [66] that in the case of the interaction of anchors with smaller heads, the detachment of the failure cone is mainly caused by concrete failure under tension (circumferential cracking) rather than under compression (the undercutting anchors modeled in this article are more likely to be included in this group of anchors). Numerical studies show that this phenomenon intensifies for rocks of lower strength. It was also observed that there are greater displacements/deformations in the contact zone with the anchor head. 

Gontarz et al. [30] and the authors of the present research have shown in previous publications [15,20] the limitations of the ABAQUS algorithm, which enable the generation of the full fracture trajectory (until reaching the free surface of the model). It is only reliable to analyze the parameters of the destruction zone in the initial range of crack development because, after this point, the algorithm cannot correctly determine the direction of crack propagation at its tip due to the appearance of the second cracking mode. As a result, the current calibration of the numerical model set by previous reviewers based on the results of field research is not very precise. Regarding the results of the field tests, detailed estimations were made on the relationships between, for example, the effective embedment depth or the range of detachments (the radius of the base of the failure cone), which were described, for example, in [5,11,21,29]. A potential correction of the calculation algorithm made by the manufacturer in the subsequent versions of the program will enable a full analysis of the issue.

## 6. Conclusions

The numerical analysis showed that the formation of radial fractures should be regarded as a natural process accompanying the action of the undercutting anchor on the rock medium (including homogeneous rock), which can lead to the fragmentation of the detached solids (in a cone-like form). The field tests confirmed the existence of a failure mechanism derived from the FEM analysis, including, in particular, the existence of the possibility of radial fractures, leading to the dismemberment of the so-called cone of destruction into finer fractions.

The general premises related to the implementation of the breakout process with the use of undercut anchors, which resulted from the results of the field tests and numerical simulations obtained so far, are as follows:

- The strong dependence of the range of detachments, and thus the volume of the detached rocks, depends to a large extent on the strength parameters of the rock. The greater the compressive and tearing strength, the smaller the range, and vice versa.
- Due to the fact that the range of breakouts (resulting from the angle of the so-called failure cone *α*) strongly depends on the effective anchorage depth (which decreases rapidly with the increase in this depth), it is rational to carry out breakouts with depths less than 100 mm.

The experimental studies and numerical simulations presented herein showed the greater susceptibility of rocks to the fragmentation of the destruction zone (through the development of radial fractures) in the case of weaker rocks and smaller anchoring depths. The determination of the optimal sets of technological parameters for anchoring and the geometrical parameters of the anchor head in the context of a given rock mass require further detailed research.

## Figures and Tables

**Table 1:** Material properties of the rock model: homogeneous, isotropic, and elastic with linear characteristics.

Material Model	Linear Elastic
Young’s modulus (E)	14.276 MPa
Poisson’s ratio (ν)	0.247
Tensile strength (*f*_t_)	7.74 MPa
Damage criterion	“max. principal stress”
Damage evolution	type Energy, Softening: linear
Critical fracture energy rate (*G*_fc_)	0.335 N/mm
Damage stabilization	Cohesive with viscosity coefficient = 1 × 10^−6^

**Table 2:** Material properties of the anchor model: steel, linear elastic medium, anisotropic, and homogeneous.

Material Model	Linear Elastic
Young’s modulus (E)	210,000 MPa
Poisson’s ratio (ν)	0.3

## Data Availability

Data presented in this study is available from corresponding authors upon request.
